# Preterm Premature Ruptures of Membrane and Factors Associated among Pregnant Women Admitted in Wolkite Comprehensive Specialized Hospital, Gurage Zone, Southern Ethiopia

**DOI:** 10.1155/2021/6598944

**Published:** 2021-12-30

**Authors:** Muche Argaw, Yibeltal Mesfin, Shegaw Geze, Keyredin Nuriye, Bitew Tefera, Aynamaw Embiale, Wesila Mohammed, Bogale Chekole

**Affiliations:** ^1^Wolkite University, College of Medicine and Health Science, Department of Midwifery, Wolkite, Ethiopia; ^2^Wolkite University, College of Medicine and Health Science, Department of Nursing, Wolkite, Ethiopia; ^3^Madda Walabu University, College of Health Science, Department of Midwifery, Madda Walabu, Ethiopia

## Abstract

**Introduction:**

Preterm premature rupture of membrane is the rupture of membrane before 37 weeks of gestational age. It complicates approximately 3 percent of pregnancies and leads to one-third of preterm births. It increases the risk of prematurity and leads to several other perinatal and neonatal complications, including the risk of fetal death. Although the prevalence and associated factors of preterm premature rupture of the membrane were well studied in high-income countries, there is a scarcity of evidence in Ethiopia, particularly in the study area.

**Method:**

A hospital-based cross-sectional study design was conducted from 1st June to 30th June 2021 in Wolkite comprehensive specialized hospital. One hundred ninety nine (199) pregnant women were included as study subjects using a systematic random sampling technique. Data were collected using a structured interviewer-administered questionnaire. It carried out descriptive statistical analysis and statistical tests like the odds ratio. Both bivariate and multivariate logistic regression analyses were conducted. Statistically, significant tests were declared at a level of *p* value < 0.05.

**Result:**

The magnitude of preterm premature rupture membrane is 6.6%. Having gestational diabetes mellitus (AOR = 5.99 (95% CI: 1.01, 32.97) and previous history of abortion (AOR = 5.31 (95% CI: 1.06, 26.69) were found to be significantly associated with preterm premature rupture of membrane.

**Conclusion:**

Having gestational diabetes mellitus and having a previous history of abortion were significantly associated with preterm premature rupture of membrane.

## 1. Introduction

Preterm premature rupture of the membrane is defined as the rupture of membrane before labor begins and before 37 weeks of pregnancy.

The all-inclusive burden of PPROM on maternal and neonatal mortality and dreariness have many effects, such as hospitalization, financial misfortune, in case of medicating cost and treatment, and workload of the wellbeing specialists [1, 2].

Around the world, 15 million newborns that are conveyed sometime recently 37 wks each year of this over one million infant kick the bucket 28 days of life and 35% of deaths account because of complications of preterm birth. The frequency of preterm untimely crack of film influences 3-4.5% of pregnancies with a moderately higher frequency in Africa and Asia. Prove appeared that the size of PPROM accounts in Brazil (3.3%) [3], India (2.2%) [4], China (19.2%) [5], Egypt (3.9%) [6], and Nigeria (3.3%) [7].

It increases the hazard of prematurity and leads to several other perinatal and neonatal complications, counting the hazard of fetal death [8]. Survivors had numerous lifetime complications of disability, counting mental retardation and visual and hearing problems [9].

Ethiopia has designed several policies and strategies to improve maternal health and reduce neonatal, infant, and under-5 child mortality. However, 29/1000 neonatal, 48/1000 infants, and 67/1000 underfive children mortality were recorded in 2016. Ethiopia is one of the highest maternal death rates on the globe. The most common cause for this neonatal mortality is an infection, preterm, and birth asphyxia. Factors like poor socioeconomic status, hypertension, smoking during pregnancy, and multiple pregnancies are associated with PPROM [10].

Variables related to PPROM were well considered in high-income countries, but there is no proof of adequate consider in Ethiopia, especially within the study region. Hence, this thinks about pointed to decide the predominance and related components of preterm untimely burst of layer among pregnant ladies in Wolkite comprehensive specialized hospital, southern Ethiopia. Design scientific-based intervention strategies to recognize components related and suitable intervention techniques for reducing PPROM.

## 2. Methods and Materials

### 2.1. Study Settings, Period, and Design

A facility-based cross-sectional study was conducted at the department of obstetrics and gynecology of Wolkite comprehensive specialized hospital from June 01 to June 30, 2021. The hospital is located in Wolkite town, which is 155 km far from Addis Ababa, the capital city of Ethiopia. Annually, around 1856 mothers give birth.

### 2.2. The Population of the Study

The source of the population was pregnant women admitted to the maternity, high risk, and labor ward of Wolkite University's specialized referral hospital, and the study population was all pregnant women that were admitted to the maternity, high risk, and labor wards of Wolkite comprehensive specialized referral hospital during the study period.

All pregnant women who were admitted to the obstetric wards whose gestational age > 28 wks and <37 wks of gestational age at Wolkite comprehensive specialized hospital were including criteria whereas pregnant women who were seriously ill and unable to communicate were excluding criteria from the study.

### 2.3. Sample Size Determination

The sample size was calculated using single proportion formula taking the following assumptions. *p* is proportion level, *p* = 13.67 [11], *d* is the marginal error which was 0.05, and *Z* ± *α*/2 = 1.96 corresponding to 95% confidence interval. The final sample size is calculating as

N = (za/2)^2^p(1 − p/d^2^, N = (1.96)^2^0.1367(1 − 0.1367)/(0.05)^2^, N = 181.

It includes a nonresponse rate of 10%, and the final sample size is 199.

#### 2.3.1. Sampling Technique

The systematic random sampling method occurred to the study participants from maternity wards. Based on the previous three month's statistics, the estimated average number of pregnant women who were admitted to the labor, maternity, and high-risk wards of Wolkite comprehensive specialized hospital is 412. Sampling interval (*k*th) was obtained by division of the entire pregnant women (total number of pregnant women admitted in three months (412) for the desired sample size (199)). Finally, the *k*th interval is approximately 2. The first pregnant woman was randomly selected by using the lottery method, and then, every second woman who was admitted in the ward was selected based on the *k*th interval.

*Operational Definition*. PPROM is leakage of amniotic fluid before the onset of labor and after fetal viability (>28 wks) as well as before 37 wks of gestational age (20).

### 2.4. Data Collection Tool, Procedures, and Personnel

The standard, structure, and interview administering questioners were prepared in English, and the classification data collection tool was into sociodemographic factors, obstetrics, medical history, and behavioral factors.

Data on respondents' specific questionnaires were collected by reviewing medical records and through interviewing the respondents. Chart review and interview were used to collect the data. Five midwives and two supervisors were used for data collection and supervisory activities, respectively.

Interviewer structure administered data collection formats were adapted and modified from different kinds of previously studied literature reviews.

### 2.5. Data Quality Management

Initially, the questionary was prepared in English and then translated to the local language (Amharic) and retranslated back into English by experts to evaluate its consistency.

Data collectors were trained for the necessary approaches, questioners were checked for completeness, and supervisors were taken a role to assure the reliability of the data collected by data collectors as well as the collected data were coded appropriately. The data were cleaned and analyzed, and pretest was conducted on 5% in the amount of the study participants at Atat Hospital prior to the data collection period. The reliability of questioners was checked by using Cronbach's alpha (≥0.75). The supervisors and principal investigators checked every day after data collection for their completeness and supplemented it with feedback.

### 2.6. Data Analysis

After data completeness, the questioners were coded, checked, cleared, and entered into EpiData 3.1 software and exported to SPSS software version 22 for analysis. Summary statistics such as frequency, percentage, and mean and standard deviation were computed. Initially, bivariate analysis was performed; then, multivariate analysis was carried out.

Bivariate logistic regression with a significance level of *p* < 0.25 was entered into a multivariate logistic regression model. Assumption of logistic regression model fitness was checked by using Hosmer and Lemeshow goodness of fit test statistics. Variables with a *p* value < 0.05 were considered as statically significant, and adjusted odds ratio with 95% CI was used to measure the strength of association.

## 3. Result

### 3.1. Sociodemographic Characteristics of the Respondent

The number of respondents was 197 with a response rate of 98.99%. Minimum and maximum ages of the respondent were 18 and 39 years, respectively, with a mean of 27.63 and standard deviation of 5.64 (Table 1).

### 3.2. Behavioral Work-Related and Obstetric Characteristics

The lowest and highest weights for mothers were 48 kg and 76 kg with a mean of 60.94 kg as well as a standard deviation of 8.03 kg. Among the respondents, 128 (65%), 56 (28.4%), and 13 (6.6%) of the respondents had BMI 18-24.9 kg/m^2^, 25-29 kg/m^2^, and >29 kg/m^2^, respectively. Similarly, 123 (62.4%) of the respondents have no history of lifting (Table 2).

### 3.3. The Magnitude of Preterm Premature Rupture of Membrane

**Figure 1:**
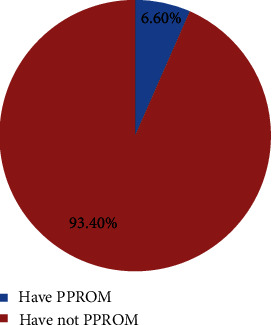
Prevalence of preterm premature rupture of membrane among pregnant women admitted in Wolkite comprehensive specialized hospital, Gurage zone, southern Ethiopia, 2021.

In this study, a total of 13 (6.6%) (95% CI: 95% CI: 3.0, 10.2) of pregnant women face rupture of membrane before 37 wks of gestation, whereas 184 (93.4%) did not face PPROM before the term of gestation (<37 wks) (Figure 1).

### 3.4. Factors Associated with PPROM

In this study, variables that have a *p* value less than 0.25 in bivariate analysis were considered as a candidate and entered into the multivariable analysis. Hence, vaginal bleeding, gestational DM, anemia, hypertension, history of abortion, and current habit of smoking were candidate variables for the multivariable analysis. Among those candidate variables having gestational DM and having a history of abortion were statistically significant in a multivariable logistic regression model.

The odds of having preterm premature rupture of membrane among pregnant women who have gestational DM were nearly six times higher as compared with those who did not have GDM (AOR: 5.99; 95% CI: 1.01, 32.97). The odds of having preterm premature rupture of membrane among pregnant women who had a history of abortion were five times higher as compared with those who did not have a history of abortion, AOR: 5.31 (95% CI: 1.06, 26.69) (Table 3).

## 4. Discussion

In our study finding, the prevalence of premature rupture of the membrane is 6.6%.

This study finding was in line with the study conducted in Kambala International University Teaching Hospital in Uganda 7.5% [12], Brazil 3.1% [3], Egypt 5.3% [6], and Nigeria 3.3% [7]. This finding was also higher than the study conducted in India 2.01% [13] and south Kerala India 0.8% [4]. The discrepancy might be due to differences in accessibility and service quality in the study area.

On the other hand, this study finding is less than the study conducted in rural Uganda (13.8%) [12], China 19.2% [14], and Ethiopia 13.67% [11]. This difference might be related to the variation in sociodemographic characteristics, socioeconomic characteristics, and type of population. Moreover, it might be related to the habit-related disease of pregnant mothers across the globe, where most of the mothers across the developed countries have developed chronic diseases.

Recent studies showed the highest governmental and other nongovernmental organizations' efforts towards the reduction of maternal mortality and the intervention towards the achievements of sustainable development goals.

Having gestational diabetes mellitus was found to be a significantly associated factor for preterm rupture of membrane. The odds of having preterm rupture of membrane among women who have gestational DM were nearly six times higher than compared with those who did not have GDM. This study finding was supported by the study conducted in CHU de Québec-University Laval [15]. In addition, this finding was also supported by a case-control study that showed that diabetes mellitus, without distinction between prepregnancy diabetes and gestational diabetes, was statistically significantly associated with preterm premature rupture of membrane [16]. This might be related to the effect of gestational diabetes mellitus in the promotion of the production of advanced glycerin end products, ligands of RAGE, a receptor implicated in this pathway.

Similarly, having a history of abortion has become a significantly associated factor for preterm premature rupture of membrane. The odds of having preterm premature rupture of membrane among mothers who have a history of abortion were five times higher than compared with those who did not have a history of abortion. This study was supported by the study conducted in rural Uganda, which revealed that the odds of the likelihood of the occurrences of preterm premature rupture of membrane among women who had a history of 3 or more abortions were 13 times higher than compared with those who have no history of abortion [12]. Similarly, a study conducted in Mekele town, Tigray, showed that women who have a history of abortion were 3 times more likely to have the occurrences of PPROM as compared with their counterparts [17]. This might be related to the weakening of the membranes secondary to the trauma that lied on the uterine wall. In addition, it may be caused by underlying infections or vascular complications which raised secondary to the abortion.

### 4.1. Limitations of the Study

Since the study was conducted in a health facility, it may not be generalized to the mothers who have not visited the health facility.

## 5. Conclusion and Recommendation

Even though the prevalence of PPROM was lower than the studies conducted in Ethiopia, a significant number of mothers still developed preterm premature rupture of membranes. In addition, having gestational DM and having a previous history of abortion were independent factors significantly associated with preterm premature rupture of membrane.

Pregnant women better prevent themselves from having an induced abortion and immediately seek a health facility if they face a spontaneous abortion. Pregnant women are better to have frequent visits to health facilities if they have been diagnosed with gestational DM. Wolkite comprehensive specialized hospitals better be organized and conduct community service activities to alleviate the preterm premature rupture of the membrane through awareness creation.

## Figures and Tables

**Table 1:** Sociodemographic characteristics of the pregnant women in Wolkite comprehensive specialized hospital, Gurage zone, southern Ethiopia, 2021.

Variables	Category	Frequency	Percentage
Age	18-20	15	7.6
	20-34	153	77.7
	34-39	29	14.7

Residence	Urban	123	62.4
	Rural	74	37.6

Educational status	Unable to read and write	78	39.6
	Only read and write	45	22.8
	Primary	44	22.3
	Secondary	20	10.2
	College and above	10	5.1

Occupational status	Housewife	95	48.2
	Student	8	4.1
	Farmer	59	29.9
	Merchant	13	6.6
	Private employee	4	2.0
	Government employee	18	9.1

Marital status	Single	8	4.1
	Married	182	92.4
	Divorced	4	2.0
	Widowed	3	1.5

The current habit of smoking	Yes	16	8.1
	No	181	91.9

Estimated monthly income (in ETB)	750-4982	123	62.4
	4983-20,000	74	37.6

**Table 2:** Obstetric-related characteristics of the pregnant women in Wolkite comprehensive specialized hospital, Gurage zone, southern Ethiopia, 2021.

Variables	Category	Frequency	Percentage
Gravidity	One	114	57.9
	2-4	48	24.4
	≥5	35	17.7

Parity	One	114	57.9
	2-4	48	24.4
	≥5	35	17.7

ANC initiation	Yes	161	81.7
	No	36	18.3

Number of visits (*n* = 161)	One	81	50.3
	Two	42	26.1
	Three	21	13.0
	Four and above	17	10.6

Abnormal vaginal bleeding	Yes	21	10.7
	No	176	89.3

Gestational DM	Yes	14	7.1
	No	183	92.9

UTI	Yes	16	8.1
	No	181	91.9

Anemia	Yes	13	6.6
	No	184	93.4

Hypertension	Yes	31	15.7
	No	166	84.3

Previous history of abortion	Yes	19	9.6
	No	178	90.4

**Table 3:** Bivariate and multivariant analysis of factors associated with preterm premature rupture of membrane among pregnant women at Wolkite comprehensive specialized hospital, Gurage zone, southern Ethiopia, 2021.

Variables	Category	PPROM		COR (95% CI)	AOR (95% CI)
		Yes	No		

Abnormal vaginal bleeding	Yes	5	16	6.56 (1.92, 22.44)	2.07 (0.26, 15.06)
	No	8	166	1	1

Gestational DM	Yes	5	11	4.72 (1.13, 19.66)	5.98 (1.01, 32.97)^∗∗^
	No	8	173	1	1

Anemia	Yes	5	10	5.22 (1.23, 22.01)	1.41 (0.12, 16.33)
	No	10	172	1	1

Hypertension	Yes	6	25	5.45 (1.69, 17.55)	2.12 (0.43, 10.38)
	No	7	159	1	1

History of abortion	Yes	5	14	7.59 (0.19,6.31)	5.31 (1.06, 26.69)^∗∗^
	No	8	170	1	1

## Data Availability

The corresponding author is responsible for data availability when reasonably requested.

## Abbreviations

ANC: Antenatal care
AOR: Adjusted odds ratio
CI: Confidence interval
ETB: Ethiopian birr
GDM: Gestational diabetes mellitus
MUAC: Mid-upper arm circumference
PPROM: Preterm premature rupture of membrane
SPSS: Statistical Package for Social Science.
